# An anticonvulsive drug, valproic acid (valproate), has effects on the biosynthesis of fatty acids and polyketides in microorganisms

**DOI:** 10.1038/s41598-020-66251-y

**Published:** 2020-06-09

**Authors:** Prapassorn Poolchanuan, Panida Unagul, Sanit Thongnest, Suthep Wiyakrutta, Nattaya Ngamrojanavanich, Chulabhorn Mahidol, Somsak Ruchirawat, Prasat Kittakoop

**Affiliations:** 1 Chulabhorn Research Institute, Kamphaeng Phet 6 Road, Laksi, Bangkok 10210 Thailand; 2 Program of Biotechnology, Faculty of Science, Chulalongkorn University, Bangkok, 10330 Thailand; 3 National Biobank of Thailand (NBT), National Science and Technology Development Agency, Thailand Science Park, Pathum Thani, 12120 Thailand; 4 National Center for Genetic Engineering and Biotechnology (BIOTEC), National Science and Technology Development Agency, 113 Thailand Science Park, Pathum Thani, 12120 Thailand; 5 Department of Microbiology, Faculty of Science, Mahidol University, Bangkok, 10400 Thailand; 6 Department of Chemistry, Faculty of Science, Chulalongkorn University, Bangkok, 10330 Thailand; 7 Chulabhorn Graduate Institute, Program in Chemical Sciences, Chulabhorn Royal Academy, Kamphaeng Phet 6 Road, Laksi, Bangkok 10210 Thailand; 8 Center of Excellence on Environmental Health and Toxicology (EHT), CHE, Ministry of Education, Bangkok, Thailand

**Keywords:** Drug discovery, Microbiology, Medical research, Chemistry

## Abstract

Valproic acid or valproate (VPA) is an anticonvulsive drug used for treatments of epilepsy, bipolar disorder, and migraine headaches. VPA is also an epigenetic modulator, inhibiting histone deacetylase, and it has been subjected to clinical study for cancer treatment. During the investigation of VPA on a metabolite profile in a fungus, we found that VPA has significant effects on the production of some fatty acids. Further exploration of VPA on fatty acid profiles of microorganisms, fungi, yeast, and bacteria, as well as representative gut microbiome, revealed that VPA could enhance or reduce the production of some fatty acids. VPA was found to induce the production of *trans*-9-elaidic acid, a fatty acid that was previously reported to have cellular effects in human macrophages. VPA could also inhibit the production of some polyketides produced by a model fungus. The present work suggests that the induction or inhibition of fatty acid biosynthesis by VPA (100 µM) in gut microbiome could give effects to patients treated with VPA because high doses of VPA oral administration (up to 600 mg to 900 mg) are used by patients; the concentration of VPA in the human gut may reach a concentration of 100 µM, which may give effects to gut microorganisms.

## Introduction

Valproic acid (VPA) or valproate, an anticonvulsive drug, has been used as drug for the treatment of epilepsy, bipolar disorder, and for the prevention of migraine headaches. VPA has side effects, for example, hepatic steatogenesis in rats^1^, and negative influence on hepatic carbohydrate and lipid metabolism^2^. There were a number of fatal cases of hyperammonemia for patients treated with VPA^3^. In mitochondria, VPA undergoes fatty acid *β*-oxidation pathway, causing toxicity due to interference with mitochondrial *β*-oxidation, and many serious inborn errors of metabolism are caused by VPA treatment^4^. VPA interferes with carnitine palmitoyl-transferase I, a key enzyme in mitochondrial fatty acid *β*-oxidation, and thus inducing hepatotoxicity and weight gain for patients under VPA therapy^5^. VPA could inhibit *N*-acetyl glutamate synthetase, and thus inhibiting urea synthesis^6,7^. VPA was found to induce abnormal autism-like behaviors in mice^8^. Recently, a number of research groups paid attention on the risk of VPA on autism spectrum disorders^9–14^. The discovery that VPA is an inhibitor of histone deacetylase, a promising anticancer drug target, has stimulated the scientific community worldwide to investigate the detailed mechanisms of VPA in various aspects^15,16^. Recently, drug repurposing of VPA has been intensively explored for the treatment of various diseases, for example, the treatment of breast cancer^17^, colon cancer associated with diabetes mellitus^18^, diffuse intrinsic pontine glioma^19^, high-fat diet-induced hypertension^20^, and HIV infection^21^. VPA also inhibits MAP kinase signaling and cell cycle progression in the yeast model^22^ and sensitizes hepatocellular carcinoma cells to proton therapy through the suppression of NRF2 activation^23^. VPA in combination with other anticancer drugs has been subjected to Phase II clinical study for cancer therapy^24^. Gut microbiome plays important role for human health and diseases, and it has gradually received attention over the past 15 years^25,26^. Many studies have revealed the functional interactions between the host (human) and gut microbiome, as well as the functions of gut microbiome in the healthy state and in certain disease states, e.g., diabetes, obesity, cancer, and liver diseases^25^. Drug-microbiome interactions have recently received attention from the scientific community. Previous works demonstrated the drug interaction with human gut microbiome, for example, atypical antipsychotic drug interacting with the gut microbiome in a bipolar disease cohort^27^. Herein we report the effects of an anticonvulsive drug VPA on the production of fatty acids and polyketides in microorganisms. Preliminary study on the effects of VPA on representative gut yeast and fungal strains is also investigated.

## Results

Effects of VPA on a fatty acid profile of microorganisms. It is known that epigenetic modulators, i.e., DNA methyltransferase and histone deacetylase inhibitors, could alter the biosynthesis of fungal metabolites, and thus changing natural product profiles with enhanced chemical diversity^28^. Many studies revealed the effectiveness of epigenetic modifiers for the production of new secondary metabolites in microorganisms, for example, proteasome-inhibitor^29,30^ and histone deacetylase inhibitor;^31–33^ this technique is collectively known as “One strain many compound” (OSMAC) approach. Previously, VPA, an inhibitor of histone deacetylase, was found to enhance tenfold-production of a fungal alkaloid^34^, fumiquinazoline C, and could change the profile of secondary metabolites^35^ and enhance antimicrobial activity of fungal extracts^36,37^. Initially, we preliminarily investigated the effect of VPA on the metabolite production of the marine fungus *Trichoderma reesei*, which normally produces only mevalonolactone as a secondary metabolite (^1^H and ^13^C NMR spectra of mevalonolactone are in Supplementary Information). The aim of this work is to use VPA, an epigenetic modulator, to enhance the production of other metabolites, possibly derived from mevalonolactone through the mevalonate pathway, the common biosynthetic pathway of terpenes and steroids. Previous works used different concentrations of VPA, e.g., 50 µM, 60 µM, 100 µM, and 500 µM, for the studies of the influence of VPA on the metabolite profile^34–37^. In the present work, we initially fed VPA with the concentrations of 100 µM and 300 µM to a culture of the marine fungus *Trichoderma reesei*, however, we found that VPA with the concentration at 300 µM or higher than 300 µM could inhibit the growth of the fungus. Therefore, we performed the experiment at the concentration of 100 µM. The present work revealed that VPA did not induce the fungus *Trichoderma reesei* to produce new terpenes and steroids, but it showed the effects on fatty acid profiles as observed from ^1^H NMR spectrum of a crude cell extract. Detailed analysis by gas chromatography (GC) revealed that VPA significantly induced the production of palmitic acid (C16:0) from 9.39% in a control (without VPA) to 19.89% (2.11 times increase) in the fungus treated with VPA, while it reduced the production of oleic acid (C18:1) from 71.51% in a control to 57.19% (1.25 times decrease) (Table 1). The amounts of palmitoleic acid (C16:1), stearic acid (C18:0), linoleic acid (C18:2), and α-linolenic acid (C18:3) of a control were relatively the same as that in the VPA treated fungus. The fungus treated with VPA (49.99%) had the total fatty acid less than a control (65.26%) (Table 1).

**Table 1:** Effect of VPA (100 µM) on fatty acid profile of the fungus *Trichoderma reesei*.

Condition	Fatty acid content (%), mean ± s.d. (n = 3)						
	C16:0	C16:1	C18:0	C18:1	C18:2	C18:3	Total fatty acid (%)
Control (without VPA)	9.39 ± 1.42^a^	0.37 ± 0.11	3.47 ± 0.58^a^	71.51 ± 6.24^a^	8.62 ± 2.04	0.06 ± 0.02	65.26 ± 6.52^b^
VPA, 100 µM	19.89 ± 2.96^a^	0.46 ± 0.11	4.73 ± 0.63^a^	57.19 ± 5.41^a^	7.22 ± 1.41	0.05 ± 0.02	49.99 ± 5.69^b^

a*p* ≤ 0.01 was selected as the minimum criterion for significance.

b*p* ≤ 0.05 was selected as the minimum criterion for significance.

As mentioned above, undesirable side effects related to fatty acid metabolism were observed in patients after treatment of VPA, for example, the influence on lipid metabolism^2^. VPA interfered with mitochondrial *β*-oxidation *via* fatty acid *β*-oxidation pathway, and thus causing serious inborn errors of metabolism after VPA treatment^4^. VPA induced hepatotoxicity and weight gain for patients because of the interference on a key enzyme for fatty acid *β*-oxidation^5^. The interferences of VPA on fatty acid metabolism in patients and our preliminary data of VPA on the fatty profile of the fungus *Trichoderma reesei* (Table 1) prompted us to investigate the effects of VPA on fatty acid profile in other microorganisms including representative gut microbiome.

Microorganisms from the culture collection of Thailand Bioresource Research Center (TBRC), Thailand, are used for this work. The first group of microorganism is fungi including *Fusarium oxysporum* TBRC4265, *Aspergillus aculeatus* TBRC2535, *Xylaria globosa* TBRC6767, *Cordyceps militaris* TBRC6930, and *Aureobasidium pullulans* TBRC4786. These fungi represent five groups; the fungus *F. oxysporum* TBRC4265 and *Aspergillus aculeatus* TBRC2535 are marine and soil fungi, respectively, while *X. globosa* TBRC6767, *C. militaris* TBRC6930, and *Aureobasidium pullulans* TBRC4786 are endophyte, entomopathogenic (insect) fungus, and epiphyte or endophyte of plants, respectively. Each fungus was grown in potato dextrose broth under shaking condition in the presence (100 µM) or absence (control) of VPA, and fatty acid profiles of individual culture are in Table 2. The marine fungus *F. oxysporum* TBRC4265 produced ten fatty acids including palmitic acid (C16:0; 29.40%), palmitoleic acid (C16:1; 0.71%), stearic acid (C18:0; 15.09%), oleic acid (C18:1; 32.93%), linoleic acid (C18:2; 19.94%), α-linolenic acid (C18:3; 0.44%), arachidic acid (C20:0; 0.65%), docosanoic acid (C22:0; 0.43%), erucic acid (C22:1; 0.09%), and lignoceric acid (C24:0; 0.33%). After feeding 100 µM of VPA to the culture of the marine fungus *F. oxysporum* TBRC4265, the fungus completely stopped the production of palmitoleic acid (C16:1), α-linolenic acid (C18:3), arachidic acid (C20:0), and lignoceric acid (C24:0) (Table 2). However, VPA significantly enhanced the production of some fatty acids by the marine fungus *F. oxysporum*, e.g., palmitic acid (C16:0) (from 29.4% (control) to 51.79%, 1.76 times of the control), docosanoic acid (C22:0) (from 0.43% (control) to 0.97%, 2.25 times of the control), and erucic acid (C22:1) (from 0.09% (control) to 1.44%, 16.0 times of the control). In contrast, VPA reduced the production of linoleic acid (C18:2) from 19.94% in control to 5.27% in the VPA treated culture of *F. oxysporum*, accounting for 3.78 times less than control (Table 2). The soil fungus *Aspergillus aculeatus* TBRC2535 did not produce α-linolenic acid (C18:3), however, after feeding 100 µM of VPA, the fungus was induced to produce α-linolenic acid 1.27% (Table 2). VPA enhanced the production of certain fatty acids by *Aspergillus aculeatus* TBRC2535, e.g., linoleic acid (C18:2) increased from 2.80% (control) to 27.20%, 9.71 times of the control) and lignoceric acid (C24:0) increased from 6.88% (control) to 11.30%, 1.64 times of the control). However, the reduction of palmitic acid (C16:0) from 41.52% (control) to 22.01% (1.88 times less than the control), palmitoleic acid (C16:1) from 0.28% (control) to 0.14% (2.00 times less than the control), stearic acid (C18:0) from 17.29% (control) to 8.81% (1.96 times less than the control), and arachidic acid (C20:0) from 0.84% (control) to 0.24% (3.5 times less than the control) was observed in the VPA treated culture of *Aspergillus aculeatus* (Table 2). VPA was found to inhibit the production of arachidic acid (C20:0) in the endophytic fungus *X. globosa* TBRC6767, 0.39% of arachidic acid (C20:0) found in the control, but none found in the VPA treated culture (Table 2). VPA also inhibited the production of lignoceric acid (C24:0) in the insect fungus *C. militaris* TBRC6930, 0.28% of lignoceric acid produced in the control culture, but none detected in the VPA treated culture (Table 2). In contrast, VPA did not have significant effects on the fatty acid profile of the fungus *Aureobasidium pullulans* TBRC4786 (Table 2), which is an epiphyte or endophyte of plants. The total fatty acid of *F. oxysporum* was reduced from 45.33% to 9.85% (4.60 times less than the control), while those of *Aspergillus aculeatus* and *Aureobasidium pullulans* were increased from 12.73% to 29.13% (2.28 times more than the control) and from 27.62% to 40.16% (1.45 times more than the control) (Table 2). VPA did not give significant effects on the total fatty acid of *X. globosa* and *C. militaris*. Overall, these results indicated that VPA could stop or enhance the production of certain fatty acids in fungi, and it also had effects on the total fatty acid of some fungi.

**Table 2:** Effect of VPA (100 µM) on fatty acid profile of fungi.

Fungi, condition	Fatty acid content (%), mean ± s.d. (n = 3)										
	C16:0	C16:1	C18:0	C18:1	C18:2	C18:3	C20:0	C22:0	C22:1	C24:0	Total fatty acid (%)
*Fusarium oxysporum*, control (without VPA)	29.40 ± 4.91^a^	0.71 ± 0.06^a^	15.09 ± 3.82	32.93 ± 4.39^b^	19.94 ± 3.45^a^	0.44 ± 0.05^a^	0.65 ± 0.18^a^	0.43 ± 0.11^a^	0.09 ± 0.02^a^	0.33 ± 0.10^a^	45.33 ± 4.84^a^
*F. oxysporum*, 100 µM of VPA	51.79 ± 5.14^a^	0.00^a^	20.29 ± 3.29	20.24 ± 2.17^b^	5.27 ± 0.60^a^	0.00^a^	0.00^a^	0.97 ± 0.09^a^	1.44 ± 0.07^a^	0.00^a^	9.85 ± 2.53^a^
*Aspergillus aculeatus*, control (without VPA)	41.52 ± 1.61^a^	0.28 ± 0.03^a^	17.29 ± 2.11^a^	29.46 ± 3.93	2.80 ± 0.13^a^	0.00^a^	0.84 ± 0.13^a^	0.93 ± 0.08^b^	0.00	6.88 ± 0.54^a^	12.73 ± 1.56^a^
*A. aculeatus*, 100 µM of VPA	22.01 ± 1.85^a^	0.14 ± 0.04^a^	8.81 ± 1.16^a^	27.83 ± 5.33	27.20 ± 4.36^a^	1.27 ± 0.10^a^	0.24 ± 0.01^a^	1.20 ± 0.08^b^	0.00	11.30 ± 1.55^a^	29.13 ± 2.49^a^
*Xylaria globosa*, control (without VPA)	20.98 ± 1.83	0.00	21.57 ± 2.88	33.77 ± 1.61^b^	21.31 ± 3.46	0.00	0.39 ± 0.05^a^	0.00	0.00	1.98 ± 0.14^a^	47.08 ± 6.00
*X. globosa*, 100 µM of VPA	20.82 ± 4.50	0.00	22.36 ± 2.29	30.2 ± 1.37^b^	24.08 ± 3.41	0.00	0.00^a^	0.00	0.00	2.55 ± 0.13^a^	48.67 ± 8.71
*Cordyceps militaris*, control (without VPA)	36.38 ± 3.27	0.54 ± 0.08^b^	7.84 ± 0.63	36.94 ± 1.42^b^	18.03 ± 2.60	0.00	0.00	0.00	0.00	0.28 ± 0.06^a^	61.55 ± 2.23
*C. militaris*, 100 µM of VPA	31.85 ± 4.45	0.74 ± 0.09^b^	6.56 ± 1.03	41.97 ± 2.45^b^	18.88 ± 2.81	0.00	0.00	0.00	0.00	0.00^a^	58.45 ± 0.57
*Aureobasidium pullulans*, control (without VPA)	29.6 ± 2.01	0.70 ± 0.05^b^	9.05 ± 0.67^b^	41.14 ± 1.17	17.36 ± 1.39	0.00	0.00	0.00	0.00	2.15 ± 0.11	27.62 ± 3.65^b^
*A. pullulans*, 100 µM of VPA	29.08 ± 0.70	0.52 ± 0.05^b^	11.94 ± 0.79^b^	41.29 ± 0.52	15.08 ± 0.14	0.00	0.00	0.00	0.00	2.08 ± 0.24	40.16 ± 5.01^b^

^a^*p* ≤ 0.01 was selected as the minimum criterion for significance (for the same fatty acid and the same fungus with or without VPA).

^b^*p* ≤ 0.05 was selected as the minimum criterion for significance (for the same fatty acid and the same fungus with or without VPA).

Next, we tried to investigate the effects of VPA on the fatty acid profile of other microorganisms, e.g., yeast and bacteria (Tables 3 and 4). The yeasts, *Saccharomyces cerevisiae* TBRC1563, *Candida utilis* TBRC360, and *Lachancea thermotolerans* TBRC4347 were used as model microorganisms (Table 3); these strains are normally used in food and beverage production. The bacteria *Pediococcus acidilactici* TBRC7580, *Bacillus amyloliquefaciens* TBRC293, and *Acetobacter cerevisiae* TBRC6687, were used as model bacteria (Table 4). *P. acidilactici* and *A. cerevisiae* are normally used in fermented dairy products (e.g., yoghourt production) and meat (e.g., Thai fermented pork sausage or “Naem” in Thai), while *B. amyloliquefaciens* is a known source of α-amylase for the starch hydrolysis in food industry. As shown in Table 3, VPA completely stopped the production of α-linolenic acid (C18:3) in the yeasts, *S. cerevisiae* and *C. utilis*, and it also stopped the production of palmitoleic acid (C16:1) in *C. utilis*. Elevated levels of palmitic acid (C16:0) from 29.03% to 47.16% (increased by 1.62 times) and stearic acid (C18:0) from 6.60% to 10.91% (increased by 1.65 times) were observed in the VPA treated culture of *C. utilis*, while decreased level of linoleic acid (C18:2) from 15.86% to 7.72% (decreased by 2.05 times) was found in the VPA treated culture of *C. utilis* (Table 3). Moreover, the total fatty acid of the yeast, *C. utilis*, decreased from 24.35% to 15.62% (decreased by 1.55 times) was observed in the VPA treated culture of *C. utilis* (Table 3). In contrast, VPA did not have notable effects on the fatty acid profile and the total fatty acid of the yeast *L. thermotolerans* (Table 3). This result clearly showed that VPA had significant effects, both on the fatty acid profile and the total fatty acid, toward the yeast *C. utilis*. Interestingly, the yeast, *C. utilis*, was found as gut microbiome in pediatric patients with inflammatory bowel disease^38^. The effects of VPA on fatty acid profile of bacteria are shown in Table 4; VPA completely inhibited the production of lignoceric acid (C24:0) in the bacterium, *P. acidilactici*, and it also inhibited the production of oleic acid (C18:1) and arachidic acid (C20:0) in the bacterium, *A. cerevisiae*. Both *P. acidilactici* and *A. cerevisiae* are used in fermented dairy products and fermented meat (Thai sausage). The level of palmitoleic acid (C16:1) in the VPA treated culture of *B. amyloliquefaciens* was decreased from 9.72% to 4.41%, accounting for 2.2 times less than the control (Table 4).

**Table 3:** Effect of VPA (100 µM) on fatty acid profile of yeast.

Yeast, condition	Fatty acid content (%), mean ± s.d. (n = 3)						
	C16:0	C16:1	C18:0	C18:1	C18:2	C18:3	Total fatty acid (%)
*Saccharomyces cerevisiae*, control (without VPA)	15.25 ± 2.81	27.98 ± 2.17	7.52 ± 1.26	39.31 ± 2.11	7.96 ± 2.73	1.97 ± 0.21^a^	39.68 ± 6.09
*S. cerevisiae*, 100 µM of VPA	14.80 ± 1.54	30.42 ± 4.63	6.37 ± 1.38	41.13 ± 2.75	7.28 ± 1.83	0.00^a^	41.39 ± 8.34
*Candida utilis*, control (without VPA)	29.03 ± 3.40^a^	0.35 ± 0.09^a^	6.60 ± 1.17^b^	46.89 ± 4.82^b^	15.86 ± 1.50^a^	1.28 ± 0.08^a^	24.35 ± 2.29^a^
*C. utilis*, 100 µM of VPA	47.16 ± 4.31^a^	0.00^a^	10.91 ± 1.85^b^	34.21 ± 2.92^b^	7.72 ± 0.36^a^	0.00^a^	15.62 ± 1.84^a^
*Lachancea thermotolerans*, control (without VPA)	22.04 ± 2.89	25.52 ± 3.07	7.53 ± 1.52	15.65 ± 0.84^a^	27.30 ± 1.73	1.97 ± 0.25	39.22 ± 6.39
*L. thermotolerans*, 100 µM of VPA	22.10 ± 2.78	24.63 ± 0.70	7.35 ± 0.98	19.64 ± 0.71^a^	24.65 ± 3.22	1.64 ± 0.19	39.29 ± 2.88

^a^*p* ≤ 0.01 was selected as the minimum criterion for significance (for the same fatty acid and the same yeast with or without VPA).

^b^*p* ≤ 0.05 was selected as the minimum criterion for significance (for the same fatty acid and the same yeast with or without VPA).

**Table 4:** Effect of VPA (100 µM) on fatty acid profile of bacteria.

Bacteria, condition	Fatty acid content (%), mean ± s.d. (n = 3)							
	C16:0	C16:1	C18:0	C18:1	C18:2	C20:0	C24:0	Total fatty acid (%)
*Pediococcus acidilactici*, control (without VPA)	52.02 ± 4.34	0.00	39.44 ± 4.04	0.00	7.00 ± 0.44^a^	0.00	1.53 ± 0.06^a^	7.92 ± 0.48^a^
*P. acidilactici*, 100 µM of VPA	48.52 ± 1.90	0.00	40.60 ± 3.14	0.00	10.87 ± 1.27^a^	0.00	0.00^a^	3.24 ± 0.10^a^
*Bacillus amyloliquefaciens*, control (without VPA)	49.96 ± 1.35	9.72 ± 1.14^a^	30.20 ± 5.63	0.00	10.12 ± 1.49	0.00	0.00	5.09 ± 1.06
*B. amyloliquefaciens*, 100 µM of VPA	49.18 ± 1.14	4.41 ± 0.40^a^	34.12 ± 4.19	0.00	12.29 ± 2.16	0.00	0.00	3.35 ± 0.76
*Acetobacter cerevisiae*, control (without VPA)	56.17 ± 6.30	0.00	37.02 ± 6.66	0.61 ± 0.05^a^	5.76 ± 1.08	0.43 ± 0.07^a^	0.00	8.59 ± 2.79
*A. cerevisiae*, 100 µM of VPA	60.34 ± 0.65	0.00	33.27 ± 1.65	0.00^a^	6.39 ± 1.55	0.00^a^	0.00	5.50 ± 0.89

^a^*p* ≤ 0.01 was selected as the minimum criterion for significance.

Recently, gut microbiome has received attention worldwide^25,26^. Therefore, we investigated the effects of VPA drug on fatty acid profile of certain representative gut microbiome, e.g., fungi and yeast. Representative gut fungi are *Penicillium shearii* TBRC2865, *Phialemonium* sp. TBRC4709, *Cladosporium* sp. TBRC4134, and *Aspergillus flavipes* BCC28681; the genera *Penicillium*, *Phialemonium*, *Cladosporium*, and *Aspergillus* are the most prevalent in human gut^39–41^. VPA could enhance the production of linoleic acid (C18:2) from 12.46% (control) to 23.60% (increase 1.89 times) in the fungus *P. shearii* TBRC2865, however, it reduced the production of linoleic acid (C18:2) from 14.85% to 7.47% (1.98 times) in the fungus *Phialemonium* sp. (Table 5). VPA reduced the production of stearic acid (C18:0) from 17.62% (control) to 9.90% (1.77 times) in *Cladosporium* sp. TBRC4134, but it enhanced the production of stearic acid (C18:0) in *A. flavipes* BCC28681 from 8.70% (control) to 16.09% (1.85 times) (Table 5). Other changes were the reduction of docosanoic acid (C22:0) in *P. shearii* TBRC2865 from 1.30% (control) to 0.67% (1.94 times); and increase of arachidic acid (C20:0) from 0.24% (control) to 1.77% (7.3 times), docosanoic acid (C22:0) from 0.24% (control) to 2.10% (8.75 times), and lignoceric acid (C24:0) from 0.29% (control) to 1.19% (4.1 times) in the fungus *A. flavipes* BCC28681 (Table 5). Three representative gut yeasts were *Candida catenulata* TBRC223, *Candida butyri* TBRC221 (syn. *Candida aaseri*), and *Saccharomyces ludwigii* TBRC2149; the genera *Candida* and *Saccharomyces* are commonly found as prevalent gut microbiome^42,43^, particularly the yeast *Saccharomyces cerevisiae*^44^. The yeast *C. catenulata* is one of the most prevalent species in gastrointestinal tract of turkeys^45^, while *C. butyri* is found as microbiota in green olive fermentations^46^. In the present work, the yeast *S. cerevisiae* TBRC1563 previously mentioned above would be one of the representative gut yeasts (Table 3). We found that VPA completely inhibited the biosynthesis of α-linolenic acid (C18:3) in the yeast *S. cerevisiae* TBRC1563 (Table 3). The yeast *C. utilis* was found as gut microbiome in patients with inflammatory bowel disease^38^; VPA completely stopped the production of palmitoleic acid (C16:1) and α-linolenic acid (C18:3) in *C. utilis* (Table 3). As shown in Table 6, VPA completely inhibited the biosynthesis of palmitoleic acid (C16:1) and α-linolenic acid (C18:3) in the yeast *Candida catenulata* TBRC223, while it induced the production of palmitoleic acid (C16:1) in *C. butyri* TBRC221 and *trans*-oleic acid or *trans*-9-elaidic acid (*trans*-C18:1) in *Saccharomyces ludwigii* TBRC2149. Normally, *trans*-9-elaidic acid is present in yeast and is degraded by peroxisomal multifunctional enzymes^47^. It was found that *trans*-9-elaidic acid is less toxic than its *cis* isomer, oleic acid, and that they exhibited different effects in gene expression regulation and handling of excess fatty acids in yeast^48^. Moreover, *trans*-9-elaidic acid could inhibit *β*-oxidation in human peripheral blood macrophages and increase intracellular Zn^2+^ in human macrophages^49,50^. Interestingly, the present work revealed that VPA could induce the production of *trans*-9-elaidic acid in the representative gut yeast. VPA reduced the production of oleic acid (C18:1) from 48.75% (control) to 23.63% (2.06 times) and linoleic acid (C18:2) from 5.61% (control) to 0.35% (16.02 times) in *S. ludwigii* TBRC2149 (Table 6). VPA markedly enhanced the production of the total fatty acid in the yeast *C. butyri* TBRC221 from 7.39% (control) to 14.18% (1.91 times). Although the present work has not investigated the effects of VPA on fatty acid profile of the representative gut bacteria, it is more likely that VPA may give effects on the biosynthesis of fatty acid of gut bacteria. The genus *Pediococcus* is normally found as intestinal flora of humans and animals^51^, the bacterium *Pediococcus acidilactici* TBRC7580 mentioned in Table 4 may be used as the representative gut bacterium. Normally, *P. acidilactici* is used as probiotic, and oral feeding study revealed that this bacterium can survive in gastrointestinal tract of volunteers about two weeks after feeding^52^. The present work showed that VPA completely inhibited the biosynthesis of lignoceric acid (C24:0) in *P. acidilactici* (Table 4).

**Table 5:** Effect of VPA (100 µM) on fatty acid profile of representative gut fungi.

Fungi, condition	Fatty acid content (%), mean ± s.d. (n = 3)									
	C16:0	C18:0	C18:1	C18:2	C18:3	C20:0	C21:0	C22:0	C24:0	Total fatty acid (%)
*Penicillium shearii*, control (without VPA)	24.29 ± 0.73^a^	17.09 ± 2.94	39.94 ± 4.29	12.46 ± 1.43^a^	0	1.65 ± 0.10^a^	1.61 ± 0.19^a^	1.30 ± 0.18^a^	1.66 ± 0.37	49.13 ± 3.14^b^
*P. shearii*,100 µM of VPA	16.86 ± 0.79^a^	13.37 ± 1.98	42.23 ± 3.62	23.60 ± 3.49^a^	0	1.08 ± 0.05^a^	1.02 ± 0.14^a^	0.67 ± 0.16^a^	1.17 ± 0.13	54.31 ± 1.60^b^
*Phialemonium* sp., control (without VPA)	28.73 ± 0.36^a^	8.23 ± 0.43	43.27 ± 0.92^a^	14.85 ± 0.56^a^	2.53 ± 0.05^a^	0.36 ± 0.02^a^	0.58 ± 0.09	0.84 ± 0.08^a^	0.61 ± 0.06^a^	64.44 ± 5.15
*Phialemonium* sp., 100 µM of VPA	22.16 ± 0.38^a^	7.94 ± 0.24	57.95 ± 0.48^a^	7.47 ± 0.29^a^	1.52 ± 0.06^a^	0.58 ± 0.02^a^	0.51 ± 0.09	1.05 ± 0.01^a^	0.83 ± 0.03^a^	72.17 ± 3.64
*Cladosporium* sp., control (without VPA)	35.29 ± 1.97	17.62 ± 1.74^a^	32.69 ± 1.57	12.55 ± 2.13	0.20 ± 0.05^b^	0.52 ± 0.02^a^	0.44 ± 0.14^b^	0.35 ± 0.03^a^	0.34 ± 0.06^b^	73.50 ± 3.86^a^
*Cladosporium* sp., 100 µM of VPA	38.77 ± 2.50	9.90 ± 0.81^a^	33.78 ± 2.30	15.20 ± 1.79	0.10 ± 0.02^b^	0.80 ± 0.07^a^	0.77 ± 0.11^b^	0.53 ± 0.03^a^	0.16 ± 0.07^b^	58.34 ± 1.27^a^
*Aspergillus flavipes*, control (without VPA)	21.30 ± 0.69^a^	8.70 ± 0.46^a^	42.17 ± 1.78	26.58 ± 1.10^a^	0	0.24 ± 0.03^a^	0.49 ± 0.02	0.24 ± 0.02^a^	0.29 ± 0.05^a^	72.03 ± 5.77
*A. flavipes*, 100 µM of VPA	18.34 ± 0.59^a^	16.09 ± 1.74^a^	39.13 ± 3.33	20.85 ± 1.76^a^	0	1.77 ± 0.05^a^	0.53 ± 0.03	2.10 ± 0.06^a^	1.19 ± 0.07^a^	74.77 ± 6.00

^a^*p* ≤ 0.01 was selected as the minimum criterion for significance (for the same fatty acid and the same fungus with or without VPA).

^b^*p* ≤ 0.05 was selected as the minimum criterion for significance (for the same fatty acid and the same fungus with or without VPA).

**Table 6:** Effect of VPA (100 µM) on fatty acid profile of representative gut yeast.

Yeast, condition	Fatty acid content (%), mean ± s.d. (n = 3)							
	C16:0	C16:1	C18:0	*trans*-C18:1	*cis-*C18:1	C18:2	C18:3	Total fatty acid (%)
*Candida catenulata*, control (without VPA)	31.35 ± 1.53^a^	5.54 ± 0.35^a^	15.13 ± 1.07	0	29.90 ± 1.89^a^	16.75 ± 1.70	1.33 ± 0.13^a^	15.41 ± 1.36^a^
*C. catenulata*, 100 µM of VPA	48.10 ± 2.12^a^	0^a^	15.66 ± 1.31	0	19.74 ± 1.78^a^	16.51 ± 1.06	0^a^	10.32 ± 1.12^a^
*Candida butyri*, control (without VPA)	38.52 ± 1.91^b^	0^a^	9.35 ± 0.72	0	21.69 ± 1.31	30.44 ± 1.82	0	7.39 ± 1.01^a^
*C. butyri*, 100 µM of VPA	33.84 ± 1.59^b^	1.32 ± 0.29^a^	8.87 ± 0.47	0	23.25 ± 1.49	32.72 ± 1.46	0	14.18 ± 1.12^a^
*Saccharomyces ludwigii*, control (without VPA)	13.92 ± 1.90	25.40 ± 0.63	6.32 ± 0.61^b^	0^a^	48.75 ± 1.51^a^	5.61 ± 0.16^a^	0	36.87 ± 2.20^b^
*S. ludwigii*, 100 µM of VPA	13.42 ± 1.49	27.94 ± 0.32	4.70 ± 0.46^b^	29.97 ± 2.12^a^	23.63 ± 1.13^a^	0.35 ± 0.06^a^	0	31.54 ± 1.60^b^

^a^*p* ≤ 0.01 was selected as the minimum criterion for significance (for the same fatty acid and the same yeast with or without VPA).

^b^*p* ≤ 0.05 was selected as the minimum criterion for significance (for the same fatty acid and the same yeast with or without VPA).

**Figure 1:**
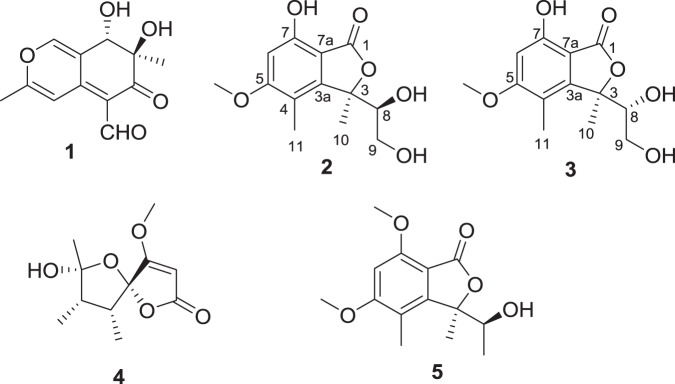
Structures of compounds 1–5 isolated from the endophytic fungus *Dothideomycete* sp.

It is known that the enzymes responsible for the biosynthesis of fatty acids and polyketide natural products share a great deal of similarities^53^. Since the above data demonstrated that VPA has effects on the production of fatty acids, we envisage that VPA may have effects on the biosynthesis of polyketide natural products because fatty acid synthases and polyketide synthases have similar catalytic elements, for example, the use of common precursors and catalytic roles. Therefore, we investigated the effects of VPA on the production of polyketide natural products using the endophytic fungus *Dothideomycete* sp., which is a known source of polyketides in our laboratory^54–56^. The fungus *Dothideomycete* sp. was previously found to produce a tricyclic polyketide, and other polyketides such as azaphilone, hybrid azaphilone-pyrone, calbistrin, and isochromanone, and it produced a large amount of austdiol (1) as the major azaphilone^54–56^. However, in the present study, in addition to austdiol (1), the fungus *Dothideomycete* sp. produced other types of polyketide, i.e., known isobenzofuranone polyketides 2, 3, and 5, and a polyketide 4 (Fig. 1). Compounds 2 and 3 were previously obtained as a mixture (1:1 ratio), which could not be separated by C18 reversed phase HPLC^57^. In the present work, compounds 2 and 3 were obtained after repeated HPLC separation; structures of both compounds were elucidated by analysis of 1D and 2D NMR spectra, as well as by data comparison with those published^57^. The absolute configuration of isobenzofuranones (e.g., 2, 3, and 5) was well established by the modified Mosher’s method and CD spectra after derivatization^58^, as well as by X-ray analysis for quadricinctone A (5)^59^. The isomer with 3 *R* and 8 *S* (e.g., 2 and 5) had negative values, while that with 3 *R* and 8 *R* (e.g., 3) had positive values^58,59^. Therefore, a polyketide 2 had 3 *R* and 8 *S* configuration because of negative optical rotation, [α]^27.1^_D_ -38.1 (*c* = 0.22, CHCl_3_), while a polyketide 3 had 3 *R* and 8 *R* configuration with a positive optical rotation, [α]^27.1^_D_ + 38.9 (0.25, CHCl_3_). ^1^H and ^13^C NMR data and NMR spectra of 2 and 3 are in Supplementary Information. Compound 4 was a derivative of papyracillic acid, previously isolated from the fungus *Ascochyta agropyrina* Var. *nana*^60^.

**Figure 2:**
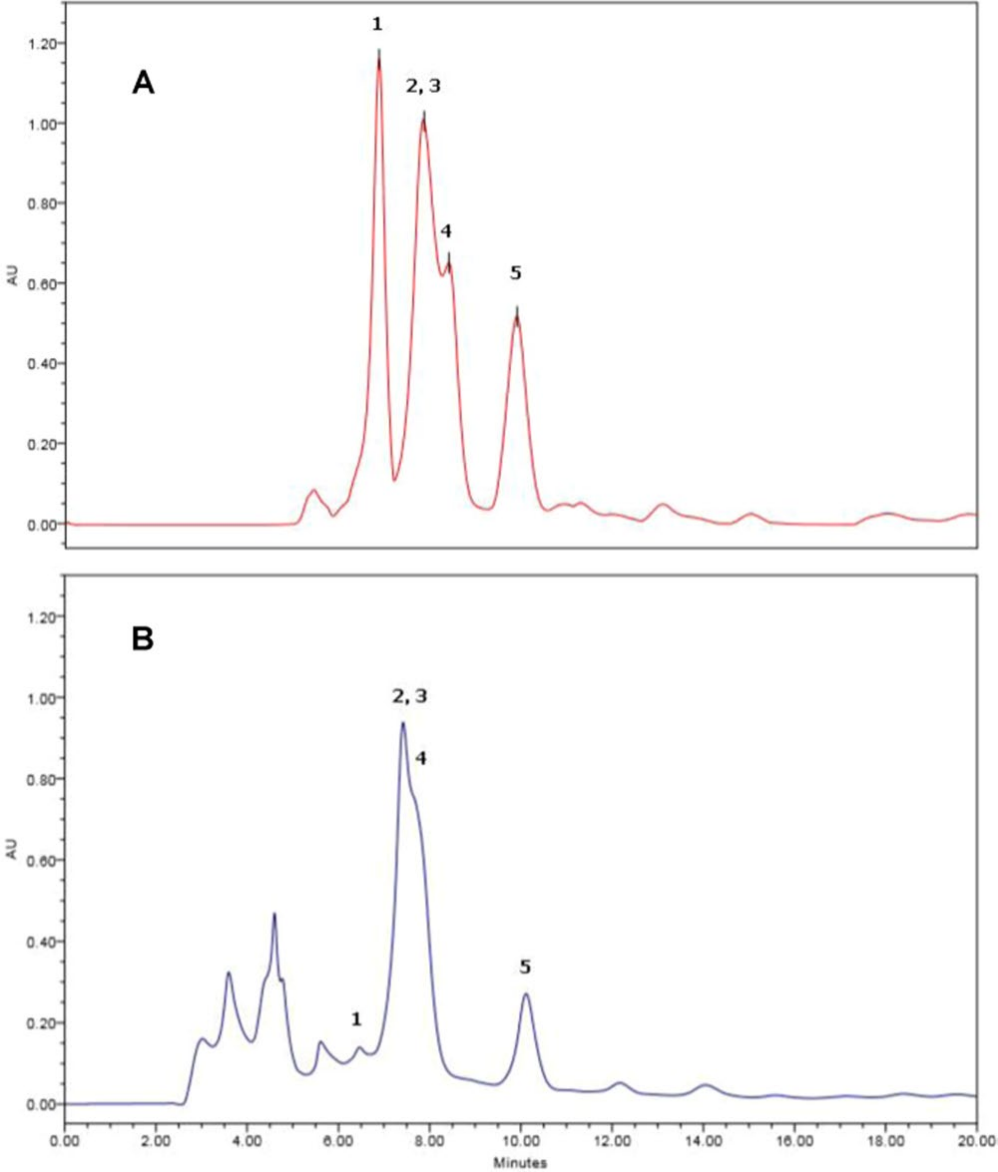
Metabolite profile of the fungus *Dothideomycete* sp. (**A**) a control culture and (**B**) VPA (100 μM) treated culture. HPLC conditions: C18 reversed column, a solvent system of MeOH:H_2_O (60:40), and UV detector set at 254 nm. 1 = austdiol (**1**), 2 = isobenzofuranone polyketide **2**, 3 = isobenzofuranone polyketide **3**, 4 = a derivative of papyracillic acid (**4**), and 5 = quadricinctone A (**5**); AU = Absorption unit.

The endophytic fungus *Dothideomycete* sp. was cultivated in 100 μM of VPA, and the comparison of the metabolite profile by HPLC analysis between the VPA treated culture and the control (without an addition of VPA) was investigated (Fig. 2). As shown in Fig. 2A, HPLC chromatogram of the control fungal culture showed peaks at retention time (*t*_R_) of 6.5 min for austdiol (**1**), at *t*_R_ of 7.4 min for compounds **2** and **3**, at *t*_R_ of 8.0 min for compound **4**, and 10.2 min for quadricinctone A (**5**). HPLC chromatogram of VPA treated culture of the fungus *Dothideomycete* sp. is shown in Fig. 2B, showing a marked reduction (>90%) of the polyketide austdiol (**1**) and *ca* 50% reduction of quadricinctone A (**5**). However, the amounts of polyketides **2**, **3**, and **4** were not affected by VPA. This experiment demonstrated that VPA could affect the production of certain fungal polyketides.

## Discussion

It is known that one of the side effects of VPA for patients is on lipid metabolism^2^; VPA interferes *β*-oxidation pathway of fatty acids, and thus causing toxicity. The negative impact of VPA treatment on serious inborn errors of metabolism is well documented^4^. VPA induced hepatotoxicity and weight gain for patients because it interferes carnitine palmitoyl-transferase I, a key enzyme in fatty acid *β*-oxidation^5^. The present work revealed that an anticonvulsive drug, VPA, has effects on the fatty acid profile of fungi, bacteria, and yeast, suggesting that this drug affects the biosynthesis of fatty acids in microorganisms. Normally, oral administration of VPA at doses of 10 to 15 mg/kg/day (i.e., 600 mg to 900 mg for the patient with 60 kg weight) is used for the treatment of epilepsy. However, doses of VPA for the prophylaxis of migraine headaches are 250–500 mg/day, while the treatment of manic episodes associated with bipolar disorder uses the dose up to 750 mg/day. In the present work, we found that VPA with the concentration 100 µM has effects on the biosynthesis of fatty acids in certain representative gut microbiome. It is possible that when patients taking high doses of VPA, i.e., 600 mg to 900 mg, the amount of VPA in patient gastrointestinal tract may reach at the concentration of 100 µM, which may affect the fatty acid biosynthesis in gut microbiome of patients. VPA induced abnormal autism-like behaviors in mice^8^, and many studies showed the possible risk of VPA on autism spectrum disorders^9–14^. The present work demonstrated that the biosynthesis of certain fatty acids was completely inhibited by VPA, while that of some fatty acids was induced by VPA. The present work showed that VPA could induce the production of *trans*-9-elaidic acid in *S. ludwigii* TBRC2149 (Table 6); this is worth mentioning because *trans*-9-elaidic acid was previously found to inhibit *β*-oxidation in human peripheral blood macrophages, and it could increase intracellular Zn^2+^ in human macrophages^49,50^. Therefore, the induction or inhibition of certain fatty acids by VPA drug may have direct effects to patients treated with VPA. Fatty acid metabolism has a critical role in human since it sustains balanced homeostasis and the negative perturbations that would lead to disease development^61^. The present work also demonstrated that VPA has effects on the biosynthesis of certain polyketide natural products produced by the fungus *Dothideomycete* sp.; this is because there are a great deal of similarities of the catalytic elements of fatty acid synthases and polyketide synthases. This knowledge may be applied for natural product research, aiming to diversify the polyketide structures.

In spite of the fact that VPA provides the effects on the biosynthesis of fatty acids in microorganisms such as fungi, yeast, and bacteria, the limitation of the present work is that representative gut microbiome used in this work does not well cover gut microbe community, particularly gut bacteria. We had selected certain genus of gut bacteria from the culture collection of Thailand Bioresource Research Center, however, most of them are anaerobic strains, growing under the conditions without oxygen. We tried to cultivate the selected gut bacteria representative using a homemade plastic incubator sealed with rubber, but failed to obtain cells for lipid extraction. In the present work, we could obtain cells from only one anaerobic bacterium, *Pediococcus acidilactici*, for lipid extraction; VPA was found to inhibit the production of lignoceric acid (C24:0) in the bacterium, *P. acidilactici* (Table 4). Recent study revealed that VPA and some psychotropic drugs gave effects on gut bacteria and short-chain fatty acids in rats, and VPA could decrease levels of propionate and butyrate, but enhancing the levels of isovalerate^62^. Many psychotropic drugs have substantial effects of gut microbiome^63^. The study in an animal model or a cohort study in patients that monitors the levels of fatty acids in feces and the composition of gut microbiota between the group treated with VPA and those without VPA will provide information regarding the precise effects of VPA on changes of fatty acids in gut microbiome.

Although this work employs the OSMAC approach using VPA as an epigenetic modulator to change the profile of secondary metabolites (natural products) in the fungus *Trichoderma reesei*, we propose that the effects of VPA toward the biosynthesis of fatty acids and polyketides may not through the epigenetic modulation. Previous works demonstrated that that VPA affected the fatty acid metabolism in patients^2^, and interfered with *β*-oxidation *via* fatty acid *β*-oxidation pathway^4^. Therefore, VPA may give direct effects toward fatty acids, i.e. the metabolism of fatty acids; however the epigenetic modulation on the biosynthesis of fatty acids could not be ruled out at this stage. Further study on the mechanistic insights into the cellular and molecular levels of VPA on changes of fatty acids and polyketides should be pursued.

## Methods

### Cultivation of microorganisms

Methods for cultivation of microorganisms, fungi, yeast, and bacteria are in Supplementary Information. Individual microorganisms were cultivated without an addition of VPA (control) or in the presence of VPA (100 µM). Cells of filamentous fungi were separated from broth by filtration using a filter paper, while cells of yeast and bacteria were collected by centrifuge at 8000 rpm. Cells of microorganisms were dried by freeze drying, and lipid in dried cells was extracted by hexane.

### Extraction of lipid and analysis of fatty acids

Dried cells of microorganisms were extracted twice by maceration in hexane overnight at room temperature. Crude fat extract was individually transesterified with 4% sulphuric acid in methanol^64^. A fat extract was dissolved in methanol containing 4% of sulphuric acid, and the mixture was heated at 90 °C for 1 h. Nonadecanoic acid (C19:0) was used as an internal standard. The esterified products were analyzed by a gas chromatography (GC) using a 30 m × 0.25 mm fused silica capillary column. The GC instrument was equipped with an automatic sampler and flame ionization detector (FID). The injector and detector temperatures were kept at 250 °C and 260 °C, respectively, and helium was used as a carrier gas at a linear velocity of 30 cm/s. The initial temperature for GC column at 200 °C was held for 10 min, and then increased at 20 °C/min to 230 °C, where it was held for 17 min. Individual fatty acid esters were identified based on the retention times relative to fatty acid methyl ester standards (Supelco 37 Component FAME Mix as the standard for methyl esters).

### Isolation of mevalonolactone

A crude broth extract (198 mg) of the fungus *Trichoderma reesei* was separated by Sephadex LH-20 column chromatography (CC) (size 2 × 132 cm), eluted with methanol, yielding twelve fractions (F1-F12). A fraction F8 (79 mg) was further separated by Sephadex LH-20 CC (size 1.5 × 126 cm), eluted with methanol, to give nine fractions (F8_1_-F8_9_). A fraction F8_1_ was purified by C18 reversed phase HPLC, eluted with a solvent system of MeOH:H_2_O (60:40), to give mevalonolactone (11.7 mg). ^1^H and ^13^C NMR spectra of mevalonolactone are in Supplementary Information.

### Isolation of compounds 1-5

A crude broth extract (107.6 mg) of the fungus *Dothideomycete* sp. was separated by C18 reversed phase HPLC using MeOH:H_2_O (60:40) as a mobile phase to yield compounds **1** (9.2 mg), **4** (4.8 mg), and **5** (5.7 mg), respectively. However, compounds **2** and **3** were obtained as a mixture from the first HPLC separation. Effort to separate compounds **2** and **3** had been made by repeated HPLC separation using MeOH:H_2_O (40:60) as a mobile phase, yielding compounds **2** (12.8 mg) and **3** (7.1 mg).

### Structure elucidation of fungal metabolites by spectroscopic techniques

^1^H, ^13^C, and 2D NMR spectroscopic data were obtained from on 400 MHz NMR spectrometer (^1^H at 400 MHz, ^13^C at 100 MHz), or 600 MHz NMR spectrometer (^1^H at 600 MHz, ^13^C at 150 MHz). Deuterated CDCl_3_ was used as an NMR solvent. HRMS data were obtained from ESI-TOF mass spectrometer. Data for optical rotations for compounds **2** and **3** were obtained from a polarimeter.

### Statistical analysis method

Statistical analysis of all data, three replications per each condition of an individual microorganism, was performed using the IBM SPSS Statistics 22 software, Independent-Samples *T* test method. Differences of fatty acid content between each group (control without VPA or with 100 µM of VPA) were determined by two-tailed *t* test, and data was reported as mean±s.d. with the significance set at *p* < 0.01 or at *p* < 0.05.

## Supplementary information

Supplementary information.
