# Supplementary material for: An anticonvulsive drug, valproic acid (valproate), has effects on the biosynthesis of fatty acids and polyketides in microorganisms
Source: Sci Rep. 2020 Jun 9;10:9300. doi: 10.1038/s41598-020-66251-y (PMC7283484; doi:10.1038/s41598-020-66251-y)

Supplementary Information

An anticonvulsive drug, valproic acid (valproate), has effects on the biosynthesis of fatty acids and polyketides in microorganisms

Prapassorn Poolchanuan[1,2], Panida Unagul[3,4], Sanit Thongnest[1], Suthep Wiyakrutta[5], Nattaya Ngamrojanavanich[6,*], Chulabhorn Mahidol[1,7], Somsak Ruchirawat[1,7,8], & Prasat Kittakoop[1,7,8,*]

[1] Chulabhorn Research Institute, Kamphaeng Phet 6 Road, Laksi, Bangkok 10210, Thailand

[2] Program of Biotechnology, Faculty of Science, Chulalongkorn University, Bangkok 10330, Thailand

[3] National Biobank of Thailand (NBT), National Science and Technology Development Agency, Thailand Science Park, Pathum Thani 12120, Thailand

[4] National Center for Genetic Engineering and Biotechnology (BIOTEC), National Science and Technology Development Agency, 113 Thailand Science Park, Pathum Thani 12120, Thailand

[5] Department of Microbiology, Faculty of Science, Mahidol University, Bangkok 10400, Thailand

[6] Department of Chemistry, Faculty of Science, Chulalongkorn University, Bangkok 10330, Thailand

[7] Chulabhorn Graduate Institute, Program in Chemical Sciences, Chulabhorn Royal Academy, Kamphaeng Phet 6 Road, Laksi, Bangkok 10210, Thailand

[8] Center of Excellence on Environmental Health and Toxicology (EHT), CHE, Ministry of Education, Thailand

Cultivation of microorganisms

The bacterium *Pediococcus acidilactici* was grown in MRS medium (Table S1), while *Bacillus amyloliquefaciens* was cultivated in NA medium (Table S2). *Acetobacter cerevisiae* was grown in GYP medium (Table S3). All bacterial strains were cultivated in 250 mL of culture medium supplemented with or without 100 µM of VPA. Three replications for each culture condition were carried out. *Pediococcus acidilactici* was incubated at 30 °C for 5 days under anaerobic condition, whereas the bacterial strains *Bacillus amyloliquefaciens* and *Acetobacter cerevisiae* were incubated with shaking at 30 °C for 5 days.

All yeast strains were grown in 250 mL of YM medium (Table S4) supplemented with or without 100 µM of VPA. Yeast cultures were incubated with shaking at 30 °C for 14 days, and three replications for each condition were carried out.

Fungi were grown in 250 mL of PDB medium (Table S5) supplemented with or without 100 µM of VPA, and three replications for each condition were performed. Fungal cultures were incubated with shaking at 30 °C for 21 days, except that the fungus *Cordyceps militaris* was cultivated at 25 °C for 21 days. Note that the fungus *Fusarium oxysporum* is a marine fungus, and it was cultivated in PDB medium prepared from seawater instead of deionized water.

The endophytic fungus *Dothideomycete* sp. was cultivated in 250 mL of PDB medium (Table S5) supplemented with or without 100 µM of VPA; the culture was incubated at 30 °C for 30 days under static condition. The marine fungus *Trichoderma reesei* was cultivated in the same manner as that of the endophytic fungus *Dothideomycete* sp., except that seawater was used instead of deionized water for the preparation of the medium.

Culture media

Table S1. Composition of MRS culture medium.

Ingredients	Amount
Proteose peptone No.3	10 g
Beef extract	10 g
Yeast extract	5 g
Dextrose	20 g
Polysorbate 80	1 g
Ammonium citrate	2 g
Sodium acetate	5 g
Magnesium sulfate	0.1 g
Manganese sulfate	0.05 g
Dipotassium phosphate	2 g
Deionized water	1000 mL

Table S2. Composition of NA (Nutrient medium) culture medium.

Ingredients	Amount
Peptone	5 g
Beef extract	3 g
Deionized water	1000 mL

Table S3. Composition of GYP (Glucose Yeast Peptone) culture medium.

Ingredients	Amount
Glucose	25 g
Yeast extract	3 g
Peptone	5 g
Deionized water	1000 mL

Table S4. Composition of YM (Yeast malt) culture medium.

Ingredients	Amount
Yeast extract	3 g
Malt extract	3 g
Peptone	5 g
Glucose	10 g
Deionized water	1000 mL

Table S5. Composition of PDB (Potato dextrose) culture medium.

Ingredients	Amount
Potato	200 g
Dextrose	20 g
Deionized water	1000 mL

Table S6. ^{1}H and ^{13}C NMR data (400 MHz, in $CDCl_3$) for compounds **2** and **3**.

Position	**2**		**3**	
	δ_C	δ_H, mult. (J in Hz)	δ_C	δ_H, mult. (J in Hz)
1	170.8	-	171.2	-
3	90.2	-	90.4	-
3a	148.7	-	149.4	-
4	112.7	-	112.5	-
5	165.5	-	165.3	-
6	98.5	6.44, s	98.3	6.43, s
7	156.6	-	156.4	-
7a	102.6	-	103.2	-
8	74.5	4.12, d (3.8)	73.6	4.17, d (4.3)
9	62.4	3.35, d, (8.1)	62.1	3.80, d, (8.0)
10	21.6	1.81, s	21.1	1.70, s
11	11.3	2.13, s	10.9	2.17, s
5-OMe	56.3	3.88, s	56.2	3.98, s

Supplemental Figure 1. ^{1}H NMR spectrum (acetone-d_6) of mevalonolactone

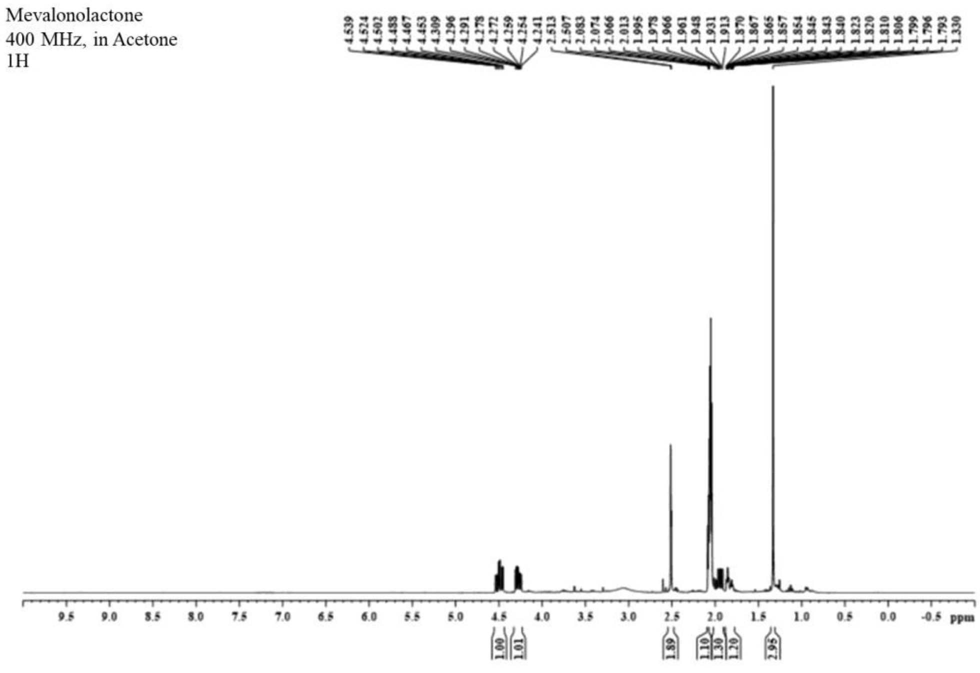

Supplemental Figure 2. ^{13}C NMR spectrum (acetone-d_6) of mevalonolactone

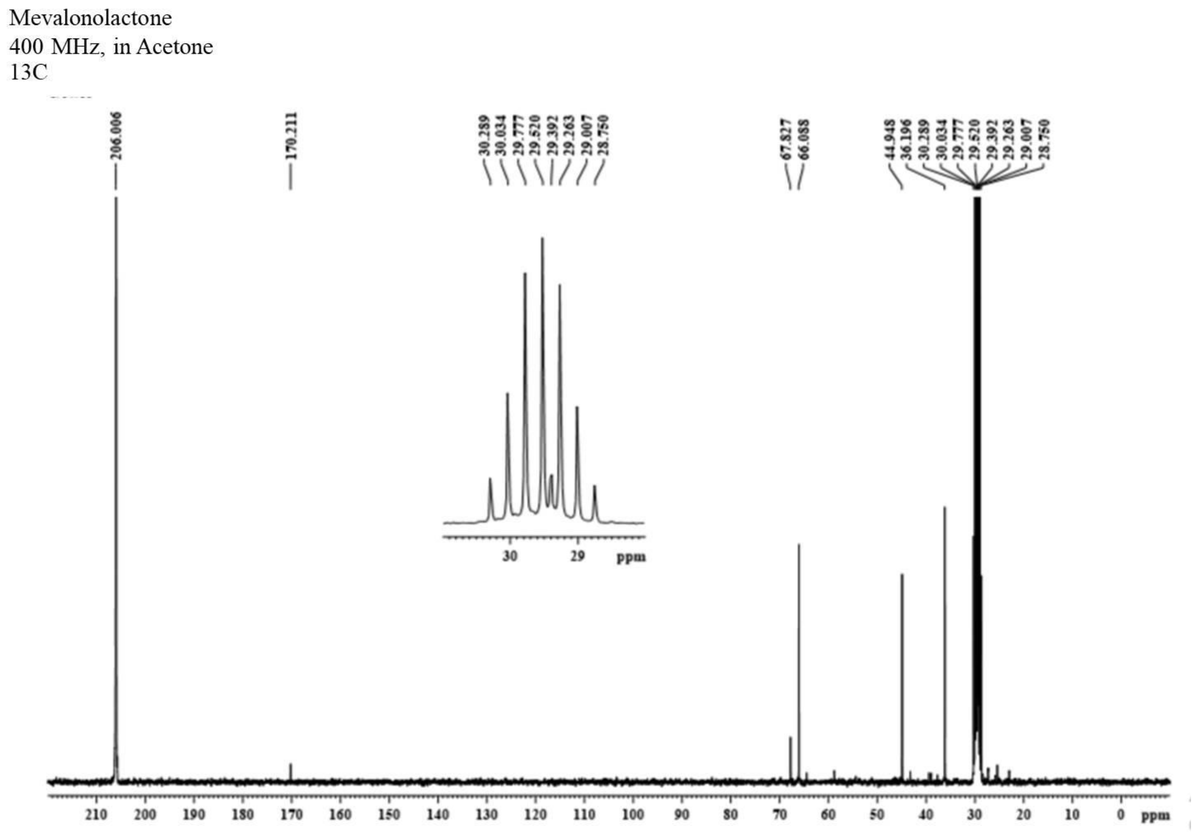

Supplemental Figure 3. ^{1}H NMR spectrum ($CDCl_3$) of compound **2**

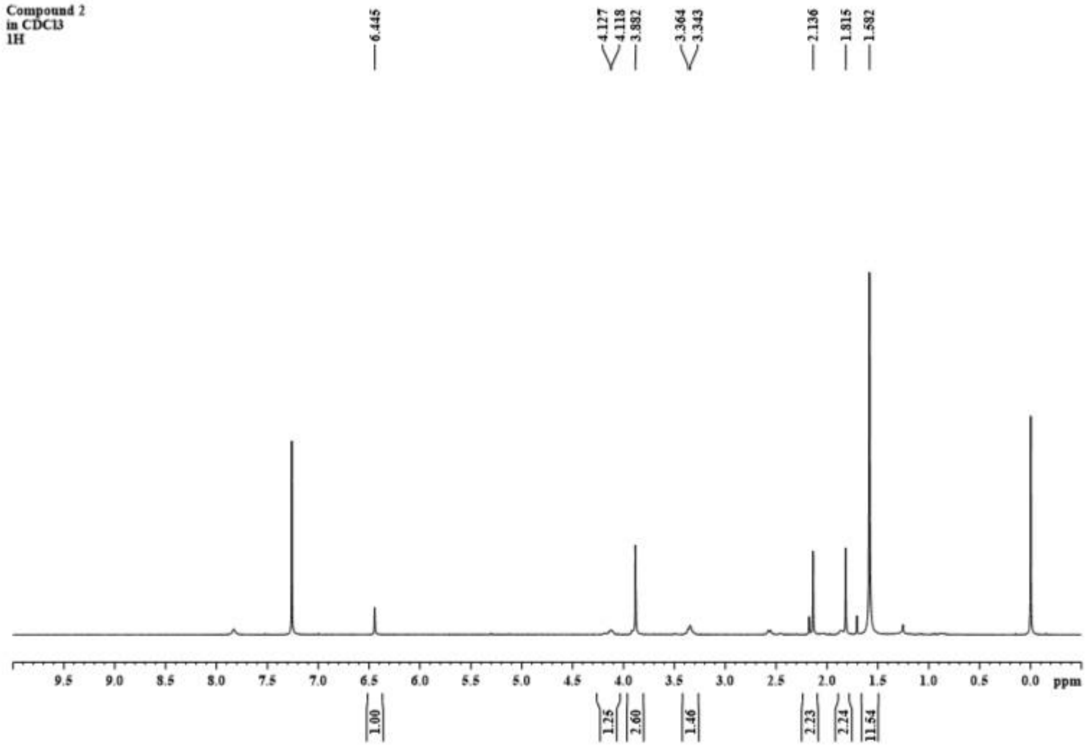

Supplemental Figure 4. ^{13}C NMR spectrum ($CDCl_3$) of compound **2**

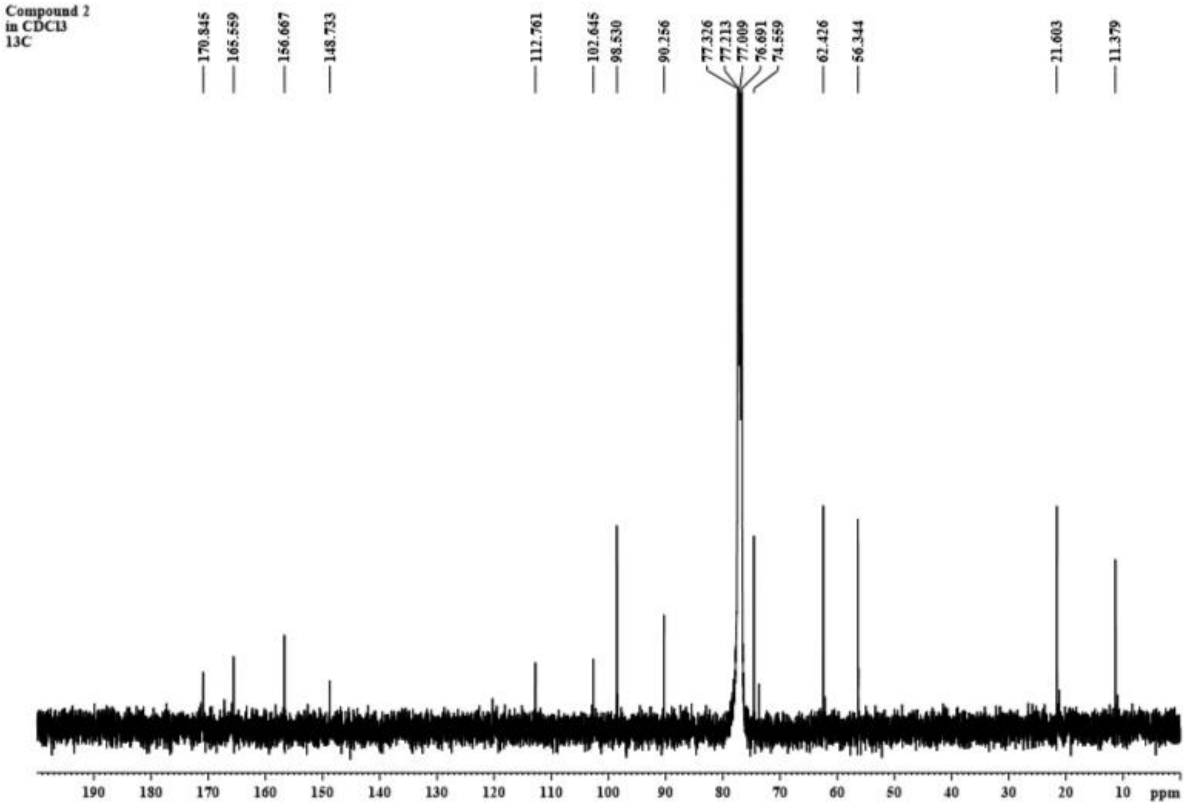

Supplemental Figure 5. ^{1}H NMR spectrum ($CDCl_3$) of compound **3**

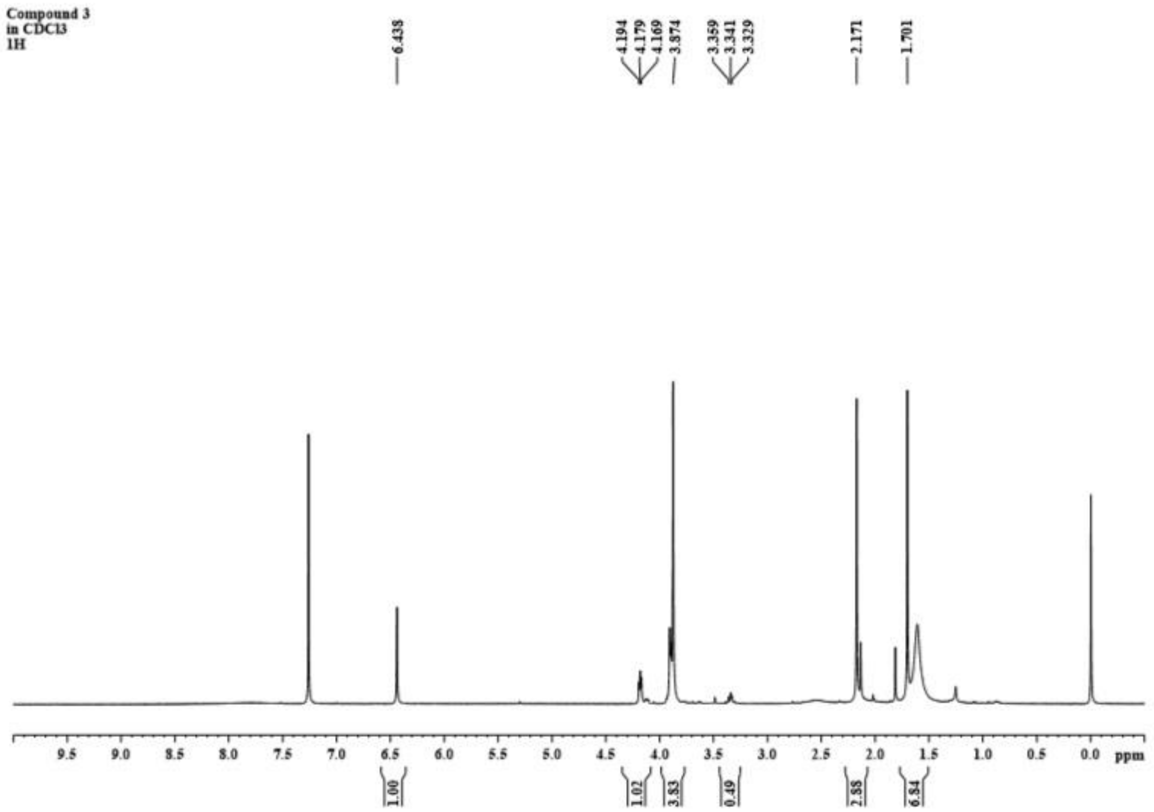

Supplemental Figure 6. ^{13}C NMR spectrum ($CDCl_3$) of compound **3**

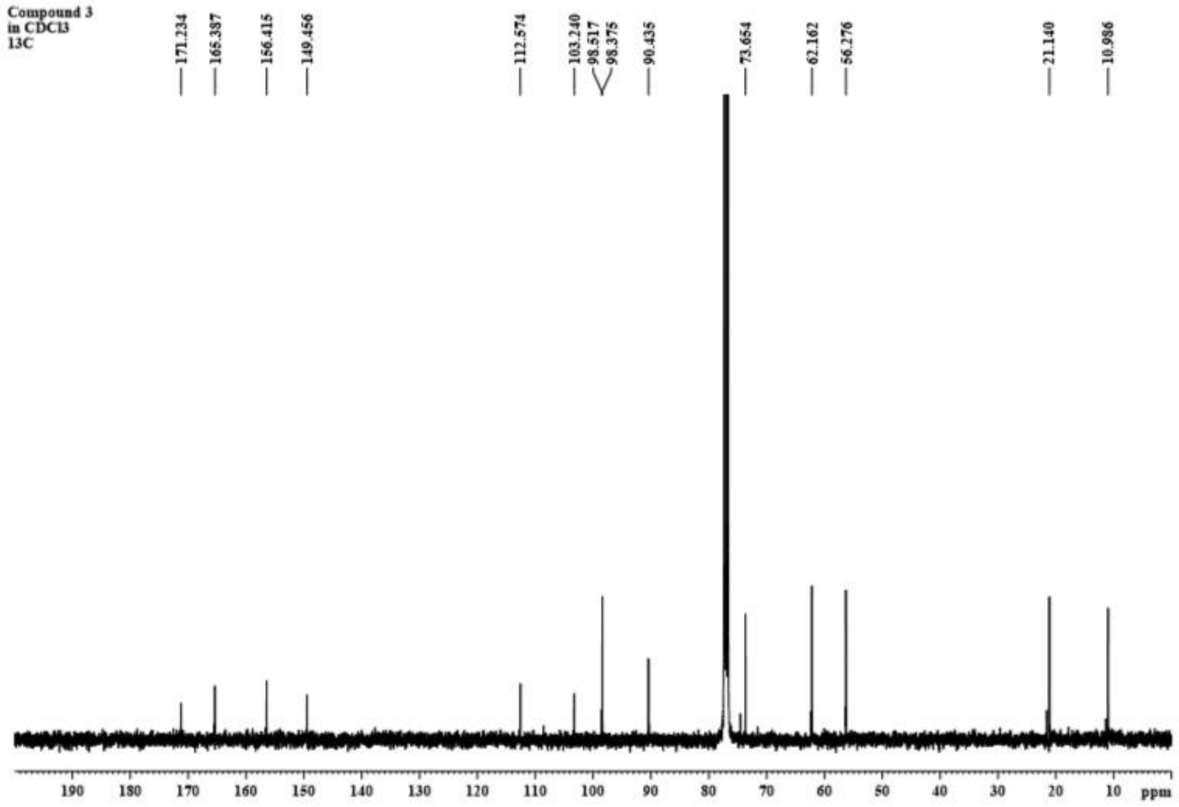

Supplement: Supplementary file 1 — Supplementary information. [file 41598_2020_66251_MOESM1_ESM.pdf]
